# Smartphone Accelerometry: A Smart and Reliable Measurement of Real-Life Physical Activity in Multiple Sclerosis and Healthy Individuals

**DOI:** 10.3389/fneur.2020.00688

**Published:** 2020-08-14

**Authors:** Yuyang Zhai, Navina Nasseri, Jana Pöttgen, Eghbal Gezhelbash, Christoph Heesen, Jan-Patrick Stellmann

**Affiliations:** ^1^Institute of Neuroimmunology and Multiple Sclerosis, University Medical Centre Hamburg–Eppendorf, Hamburg, Germany; ^2^Department of Neurology, University Medical Centre Hamburg–Eppendorf, Hamburg, Germany; ^3^Academy for Training and Career, University Medical Centre Hamburg–Eppendorf, Hamburg, Germany; ^4^APHM, Hopital de la Timone, CEMEREM, Marseille, France; ^5^Aix Marseille Univ, CNRS, CRMBM, UMR 7339, Marseille, France

**Keywords:** smartphone, multiple sclerosis, accelerometry, physical activity, ambulation

## Abstract

**Background:** Mobility impairment is common in persons with multiple sclerosis (pwMS) and can be assessed with clinical tests and surveys that have restricted ecological validity. Commercial research-based accelerometers are considered to be more valuable as they measure real-life mobility. Smartphone accelerometry might be an easily accessible alternative.

**Objective:** To explore smartphone accelerometry in comparison to clinical tests, surveys, and a wrist-worn ActiGraph in pwMS and controls.

**Methods:** Sixty-seven pwMS and 70 matched controls underwent mobility tests and surveys. Real-life data were collected with a smartphone and an ActiGraph over 7 days. We explored different smartphone metrics in a technical validation course and computed afterward correlation between ActiGraph (steps per minute), smartphone accelerometry (variance of vector magnitude), clinical tests, and surveys. We also determined the ability to separate between patients and controls as well as between different disability groups.

**Results:** Based on the technical validation, we found the variance of the vector magnitude as a reliable estimate to discriminate wear time and no wear-time of the smartphone. Due to a further association with different activity levels, it was selected for real-life analyses. In the cross-sectional study, ActiGraph correlated moderately (*r* = 0.43, *p* < 0.05) with the smartphone but less with clinical tests (rho between |0.211| and |0.337|). Smartphone data showed stronger correlations with age (rho = −0.487) and clinical tests (rho between |0.565| and |0.605|). ActiGraph only differed between pwMS and controls (*p* < 0.001) but not between disability groups. At the same time, the smartphone showed differences between pwMS and controls, between RRMS and PP-/SPMS, and between participants with/without ambulatory impairment (all *p* < 0.001).

**Conclusions:** Smartphone accelerometry provides better estimates of mobility and disability than a wrist-worn standard accelerometer in a free-living context for both controls and pwMS. Given the fact that no additional device is needed, smartphone accelerometry might be a convenient outcome of real-life ambulation in healthy individuals and chronic diseases such as MS.

## Introduction

Multiple sclerosis (MS) is the most common autoimmune disease of the central nervous system (CNS) and leads to an accumulation of disability by chronic inflammation and neurodegeneration (1). The patterns of disability are heterogeneous, but impaired mobility occurs in up to 75% of persons with multiple sclerosis (pwMS) (2) and represents one of the most disrupting physical features of MS (3). Regarding the perceptions of bodily functions, ambulation is rated as one of the three most valuable abilities (4). Besides, walking is the most frequent type of self-selected physical activity (5) and represents with over 50% of dynamic activity over a 24-h period, the primary mode of physical activity in pwMS (6). Walking impairment could cause physical inactivity, which results in physical deconditioning, and in this negative feedback mechanism, walking impairment could be driven further down (7). In the clinical setting, walking impairment can be used to monitor disability progression, and ambulatory improvement can be used as an indicator of efficacy in therapeutic trials (8). However, while the importance of the walking ability in MS is widely accepted, the ideal measurement approach is still under discussion (9).

The Extended Disability Status Scale (EDSS) is an accepted standard of disability measurement in MS and relies in its middle range mainly on walking abilities in the range between 20 and 500 m (10). However, the scale suffers for its increased variability for longer walking distances and other factors like fatigue, patient's mood, and the time the test was performed (11). EDSS also has limitations to measure small but clinically meaningful changes in ambulation, and it fails to capture the performance fluctuation over time in the natural environment (12). Standard clinical performance-based measures, such as the Timed 25-Foot Walk (T25FW), 2-Minute Walking Test (2MWT), and 6-Minute Walking Test (6MWT) (13), provide objective snapshots of the day-to-day variable ambulatory capacity (14). They may not reflect the continuous walking activity in the real-world environment due to the lack of ecological validity (15). Patient-reported outcome measures (PROMS) (e.g., 12-Items Multiple Sclerosis Walking Scale (MSWS-12) (16) or Godin Leisure-Time Exercise Questionnaire (GLTEQ) (17) are limited by recall bias and variability in self-perception of physical activity.

To that end, the total ambulatory activity undertaken in the habitual environment in performing a usual range of daily activities is recognized as the gold standard for measuring ambulatory mobility in neurological disorders (18), and there is an emerging body of research supporting the application of accelerometry for measuring physical data in MS (19, 20). ActiGraph (Pensacola, FL, USA) is one of the most common accelerometers used in research (20, 21). Associations between the output of ActiGraph (i.e., activity counts, step counts, MVPA, and sedentary time) and clinical outcomes in the free-living setting have been intensively investigated (22–24). Nevertheless, there are neither standard protocols of application of commercially available accelerometers nor standard accelerometer output—for example, estimates of energy consumption, step number, or walking speed (19, 25). The need and burden of wearing an additional device restricts its use to short-term usage and may, due to the perceived invasiveness, affect the ecological validity. Smartphone with built-in accelerometry might overcome this shortcoming and has been considered as a possible measurement for motion data. The cost and the burden of measurement are low due to a high usage rate in the general population and among pwMS and the lack of need for a further device (26). Studies in recent years supported the application of smartphones for assessing mobility and physical activity in clinical as well as in a free-living setting (27–30). However, there is a lack of studies investigating smartphone accelerometry as a putative outcome for neurological diseases such as MS.

Here, we aimed to investigate the value of built-in smartphone accelerometers as a valid outcome for disability and mobility compared to a wrist-worn ActiGraph in a representative group of pwMS compared to healthy controls in a free-living setting.

## Methods

The validation and exploration of the smartphone accelerometry were done in two steps: first, we performed a technical validation course for wear time validation and selection of outcomes. Second, we performed a cross-sectional analysis in pwMS and healthy controls with clinical outcomes, PROMS, and ActiGraph measurements. The value of an outcome metric was estimated by its discriminant ability between different disability groups (e.g., mild vs. moderate impairment) and its correlation with self-reported physical activity and clinical performance-based measures.

### Technical Validation Course

To define periods of wear time and non-wear time and to explore summary measures from the raw accelerometry data, we performed a technical validation course using 28 smartphones, Samsung Galaxy (model S4 mini) with a built-in tri-axis accelerometer. First, we collected no-wear data over 10 min while all smartphones were lying in different positions on a table. Then, the smartphones were carried by three members from the staff for investigating wear time assessments, which included sitting, standing, walking, running, and stair climbing 10 min each. Passive movements were recorded in an elevator and during a bus trip.

### Participants

Participants were recruited at the MS outpatient clinic at the University Medical Centre Hamburg–Eppendorf. The inclusion criteria for pwMS were (1) age 18–65 years, (2) a confirmed diagnosis of MS according to McDonald criteria 2010 (31), (3) an Extended Disability Status Scale (EDSS) (10) score below 6.5, and (4) no relapse in the last 30 days. The inclusion criteria for the controls were (1) not reporting disease with potential impact on mobility and (2) matching the age distribution of the sample with MS. The exclusion criteria for both samples were severe medical conditions other than MS, severe cognitive impairment, or any other condition that might relevantly compromise the use of a smartphone (e.g., very low visual acuity or severe ataxia). All participants gave written informed consent prior to any testing under this protocol, and the local ethical review board (Ärztekammer Hamburg, PVN 5001) approved the investigation.

### Procedures

After inclusion, we collected demographic data and participants filled in the following questionnaires: Godin Leisure-Time Exercise Questionnaire (GLTEQ) (17), the Frenchay Activity Index (FAI) (32), and the International Physical Activity Questionnaire (IPAQ) (33). pwMS completed also the 12-Item Multiple Sclerosis Walking Scale (MSWS-12) (16, 34). Clinic-based measures of ambulation included Five-Times Sit-To-Stand test (FTSTS), Timed 25-Foot Walk (T25FW), 2-Minute Walking Test (2MWT), 6-Minute Walking Test (6MWT), and a 3-Meter Timed Tandem Walk (TTW) (13). EDSS scoring was performed within the clinical examination by a neurologist (10).

All participants were supplied with an ActiGraph (model GT3X+) and a smartphone (Samsung Galaxy S4 mini). We asked the participants to wear the ActiGraph on the non-dominant wrist (35) and the smartphone in the habitual position like their phones for the following 7 days. They were asked to wear both devices during the entire day, except for showering, swimming, or while sleeping.

### Data Processing

All the written data, including demography, clinical performance-based measures, and PROMS were collected in an electronic case report file. The raw ActiGraph data were processed, and standard outcomes [mean vector magnitude (meanVM), daily MVPA, steps/minute] were downloaded with ActiLife 6 software version 6.13.3 (ActiGraph, Pensacola, FL USA) in 60-s epoch intervals. Non-wear time was filtered out with the Choi algorithm (36). The smartphone accelerometer data were collected via a small Android-based application, which had been developed by the Institute of Neuroimmunology and Multiple Sclerosis (INIMS). The raw accelerometer axis (*X, Y*, and *Z*) values were filed at a sampling rate of 2 Hz.

#### Selection of Smartphone Outcomes

For the selection of putative smartphone outcomes in the technical validation course, we computed and explored the following summary metrics for epochs of 60 s (same bout length as for the ActiGraph): Sum of absolute axis values (sumX, sumY, and sumZ), variance of axis values (varX, varY, and varZ), Pearson's correlations between each pair of axes (corXY, corXZ, and corYZ), sum of all absolute axis values (sumXYZ), mean absolute correlation (corXYZ), sum of absolute vector magnitude (sumVM), mean vector magnitude (VM), and mean variance of the vector magnitude (varVM). Most of the metrics reflect standard accelerometry metrics—such as the vector magnitude and sum of acceleration of selected axes (37). However, several commonly used accelerometry outcomes rely on the proper orientation in space, for example the vertical axis counts. For smartphones, such orientation-dependent metrics are not reasonable under the concept of using the patient's device in the future. Thus, we decided to explore orientation-independent metrics. We hypothesized that increasing physical activity might translate into the reduced correlation of the axes counts and increased variance of acceleration measurements.

To compare the potential metrics, the available dataset was split in a ratio of 1:1 randomly in an explorative and a validation subset. First, we used the explorative data to visually inspect boxplots of all measurements for the selection of candidates with the high discriminant ability of no-wear vs. wear time and over different activities. The potential metrics were then formally tested for discriminant abilities of wear and no-wear time by receiver operating characteristic (ROC) analyses. Finally, we validated the metrics from the explorative dataset in the validation sample and defined cutoff values for separation of wear and no-wear time. For further analysis, all accelerometry outcomes were wear-time corrected average values.

#### Statistical Analysis

For the statistical analysis, we divided the total sample into healthy controls and the pwMS. The pwMS were further divided into the following subgroups: (1) disease course (relapsing vs. progressive) representing conceptually early and late MS and (2) by EDSS <3.5 vs. ≥3.5 representing a cutoff for ambulatory impairment in MS (10) (minimal ambulatory impaired vs. ambulatory impaired). We performed descriptive statistics of the demography with mean/*SD*, median/range, or number/rates according to the nature of the data. Student's *t*-test was used to detect the differences of demography, clinical performance-based metrics, PROMS, wear time, and metrics of accelerometry within the above-mentioned groups. Associations between smartphone accelerometry and ActiGraph were first estimated by Spearman's rank-order coefficient within the groups. The most correlating metric of each accelerometer was then chosen to be tested with the clinical performance-based metrics and PROMS by Spearman's rank order. We used Mann–Whitney *U*-test to determine the ability of the accelerometers to separate between groups. In addition, we computed ROC analysis to examine the predictability of the accelerometers for disease course and severity of the disability. *P* < 0.05 was used for judging the significance level. Due to multiple comparisons, we corrected the *p*-values with the false discovery rate (FDR). All analyses were performed with statistics in *R*.

## Results

### Technical Validation and Selection of Outcomes

**Figure 1:**
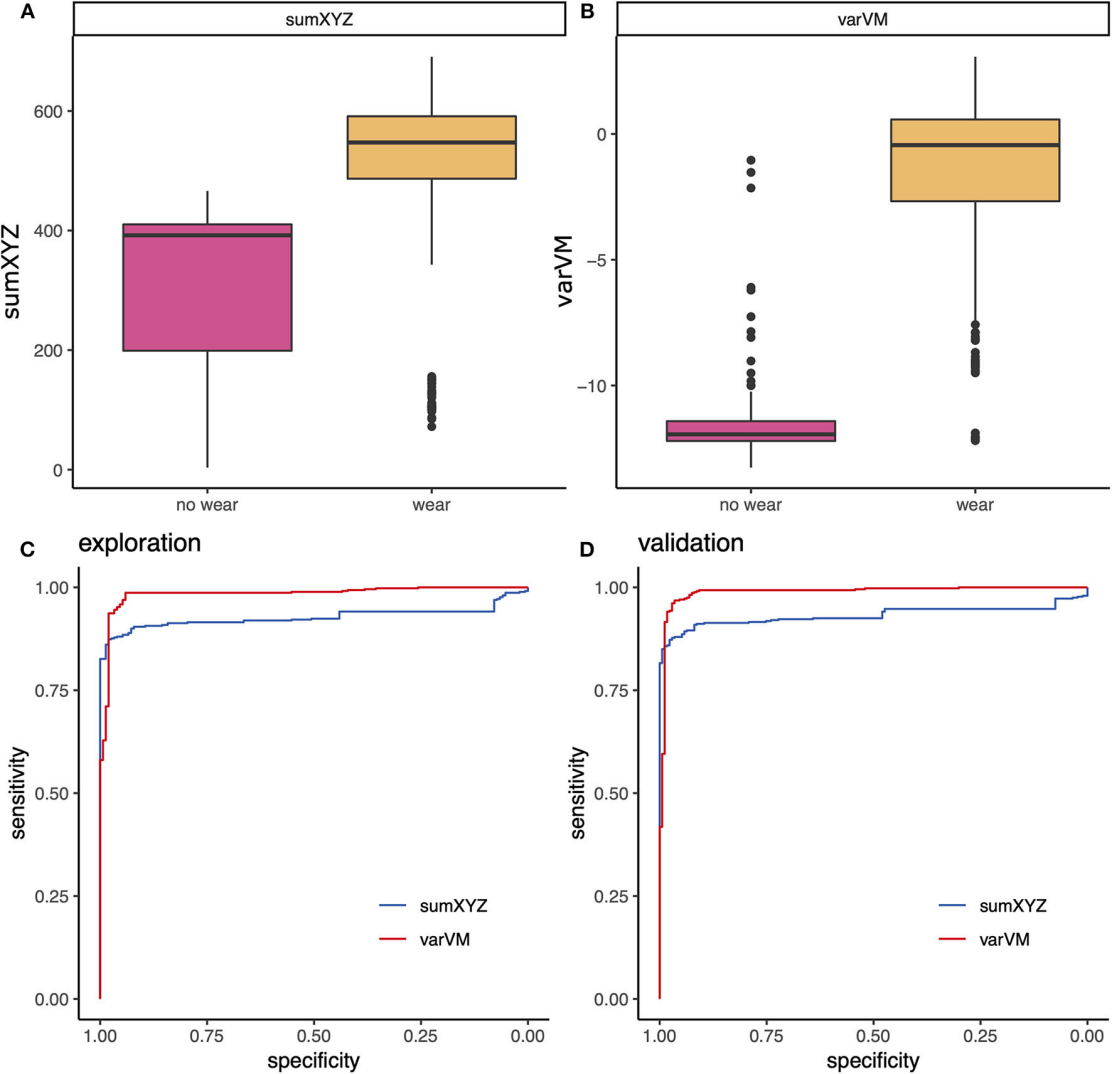
Technical validation of smartphone metrics: wear time detection. Exploration of different smartphone metrics revealed good discriminant abilities for **(A,B)** sumXYZ and varVM that could be confirmed in receiver operating characteristic (ROC) analyses of the **(C)** explorative and the **(D)** validation subset.

**Figure 2:**
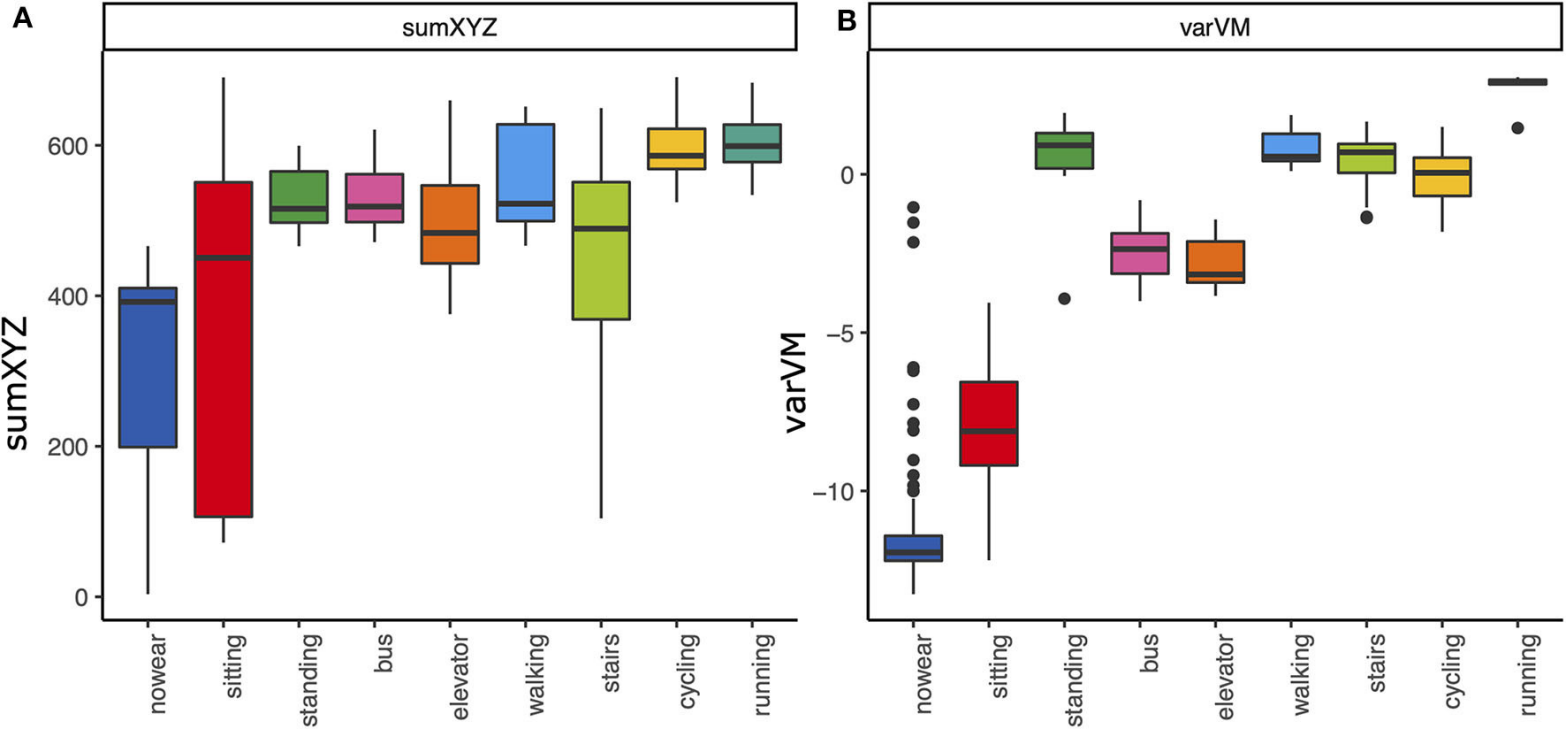
Smartphone metrics and physical activities. Boxplots of the smartphone metrics **(A)** sumXYZ and **(B)** varVM during different activities. Both metrics also tended to increase with the activity intensity.

Exploration of smartphone metrics (see Figures 1, 2 and Figures e1, e2) revealed a high sensitivity and specificity for wear time detection for sumXYZ [area under the curve (AUC) = 0.928, *p* < 0.001, accuracy = 0.901, sensitivity = 0.874, and specificity = 0.959] and several variance metrics including varVM (AUC = 0.984, *p* < 0.001, Figures 1A–C, accuracy = 0.975, sensitivity = 0.987, specificity = 0.941]. Discriminant abilities could be confirmed in the validation subset, and the AUC from the validation set did not differ from the explorative estimation for varVM (*p* = 0.507). However, varVM showed significantly higher accuracy than sumXYZ (*p* < 0.001) and was chosen for wear time detection. Moreover, both metrics tended to increase with estimated physical activity level, and we used these two outcomes for further analyses (Figures 2A,B).

### Participants and Clinical Characteristics of the Subgroups

**Figure 3:**
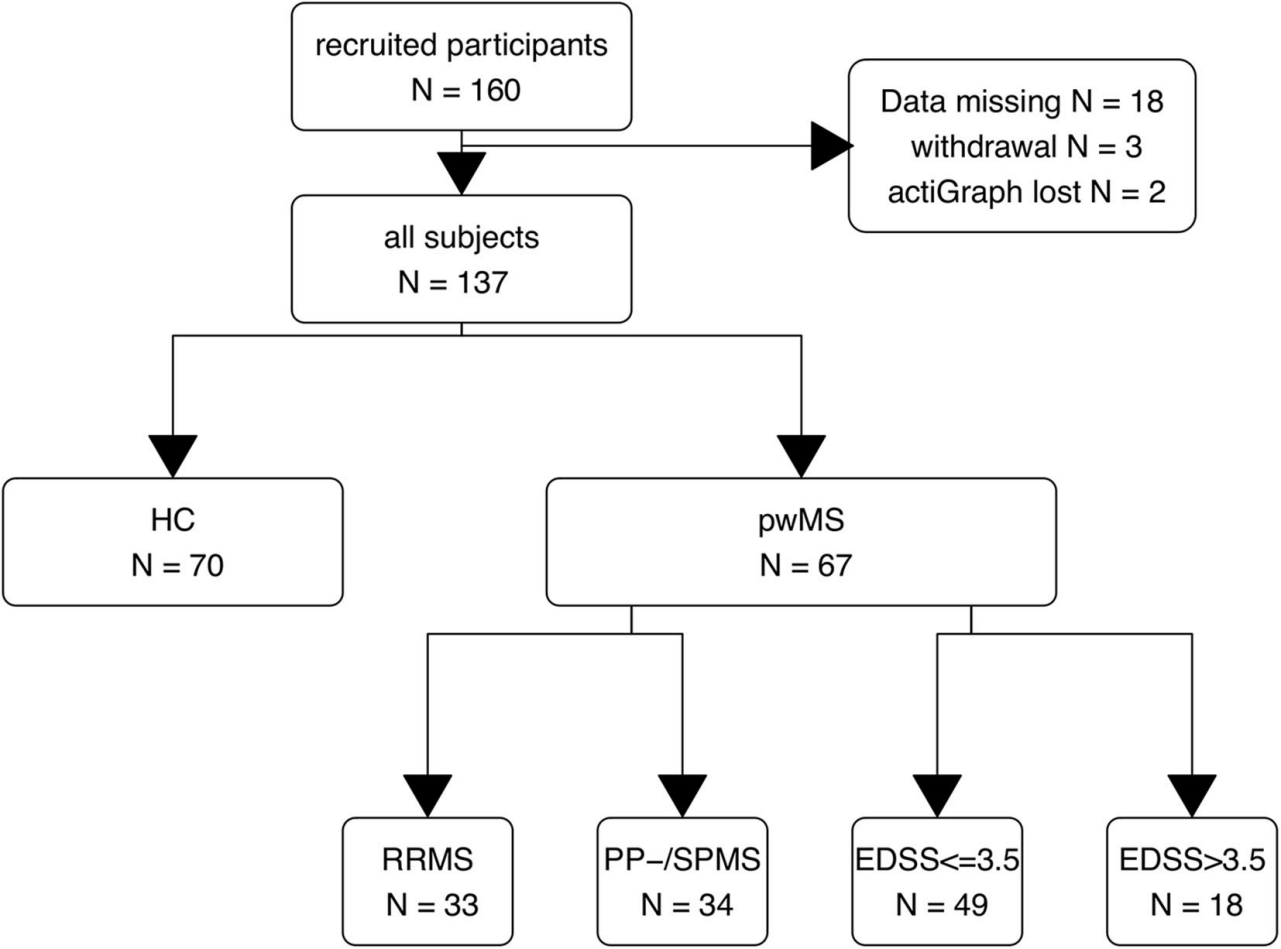
Study flow chart. HC, healthy controls; pwMS, patients with MS; RR, relapsing–remitting MS; PP-/SPMS, primary and secondary progressive MS; EDSS, expanded disability status scale (EDSS > 3 indicates walking impairment).

We included 137 subjects: 70 HC and 67 pwMS (see Figure 3). Demographic data are presented in Table 1. Patients with primary or secondary progressive MS (PP-/SPMS) were elder (49.6 vs. 35.9 years) than patients with relapsing–remitting MS (RRMS). pwMS with impaired ambulation had longer disease duration (12.9 vs. 6.4) than its comparison group. Otherwise, we observed no group differences in age, body mass index (BMI), and waist. Moreover, the median EDSS in patients with primary or secondary progressive MS (PP-/SPMS) was 1.8 higher (*p* < 0.001) than in relapsing–remitting MS (RRMS).

**Table 1:** Demographic and clinical data.

	**Control**	**pwMS**	**RRMS**	**PP-/SPMS**	**EDSS ≤ 3.5**	**EDSS > 3.5**
*N* (Sex)	70 (47F/23M)	67 (42F/25M)	34 (18F/16M)	33 (24F/9M)	49 (32F/17M)	18 (10F/8M)
		(*p* = 0.713)		(*p* = 0.155)		(*p* = 0.655)
Age (years)	41.5 ± 12.8	42.9 ± 10.9	35.9 ± 9.1	49.6 ± 7.7	41.6 ± 11.4	46.4 ± 8.4
		(*p* = 0.496)		(*p* < 0.001)		(*p* = 0.067)
Weight (kg)	71.9 ± 15.2	73.3 ± 16.8	72.2 ± 18.5	74.4 ± 15.2	74.4 ± 17.0	70.4 ± 16.6
		(*p* = 0.613)		(*p* = 0.595)		(*p* = 0.396)
BMI	24.0 ± 3.9	24.4 ± 4.7	24.5 ± 5.3	24.4 ± 4.2	24.8 ± 4.7	23.3 ± 4.7
		(*p* = 0.585)		(*p* = 0.905)		(*p* = 0.242)
Waist (cm)	89.1 ± 12.8	92.3 ± 14.8	89.9 ± 16.4	94.6 ± 12.9	93.1 ± 14.7	90.2 ± 15.5
		(*p* = 0.177)		(*p* = 0.197)		(*p* = 0.499)
Disease duration (years)		8.5 ± 8.1	6.5 ± 5.6	9.7 ± 8.6	6.4 ± 6.2	12.9 ± 9.1
				(*p* = 0.097)		(*p* = 0.012)
Median EDSS (range)		3 (1.0–6.0)	2.0 (1.0–5.5)	3.5 (2.0–6.0)	2.5 (1.0–3.5)	5.5 (4.0–6.0)

*Values represent mean ± SD, if not otherwise indicated; p-values for group comparison (patients vs. controls, RRMS vs. PP-/SPMS, and EDSS groups) based on chi-square test for rates or Student's t-test*.

*BMI, body mass index; EDSS, expanded disability status score*.

Table 2 shows the descriptive statistics of clinical tests, PROMs, and accelerometry measures of ActiGraph and smartphone. The average measurement times within 7 days were 55 h for the smartphone and 76 h for the ActiGraph, which represent an average active wear time of 7.5 and 10.9 h/day, respectively.

**Table 2:** Clinical outcomes and accelerometry data.

	**Control**	**pwMS**	**RRMS**	**PP-/SPMS**	**EDSS ≤ 3.5**	**EDSS > 3.5**
**Clinical test**						
T25FW (s)	4.0 ± 0.6	5.6 ± 2.8	4.2 ± 0.8	6.8 ± 3.5	4.7 ± 0.8	7.8 ± 3.5
		(*p* < 0.001)		(*p* < 0.001)		(*p* = 0.020)
FTSTS (s)	7.4 ± 1.7	11.7 ± 4.8	9.6 ± 1.7	13.8 ± 5.9	10.4 ± 2.7	15.8 ± 7.4
		(*p* < 0.001)		(*p* < 0.001)		(*p* = 0.039)
TTW (s)	6.6 ± 1.8	11.8 ± 5.6	8.9 ± 3.3	14.4 ± 6.2	10.4 ± 4.1	15.8 ± 7.5
		(*p* < 0.001)		(*p* < 0.001)		(*p* = 0.030)
2MWT (m)	201 ± 35	164 ± 38	179 ± 29	148 ± 40	176 ± 29	131 ± 41
		(*p* < 0.001)		(*p* = 0.002)		(*p* = 0.003)
6MWT (m)	609 ± 75	477 ± 123	530 ± 86	424 ± 132	517 ± 90	362 ± 136
		(*p* < 0.001)		(*p* < 0.001)		(*p* = 0.002)
**PROMS**						
GLTEQ	43.1 ± 27.1	25.1 ± 20.9	30.1 ± 21.6	20.6 ± 19.2	29.5 ± 20.9	13.9 ± 16.4
		(*p* < 0.001)		(*p* = 0.105)		(*p* = 0.014)
FAI	34.6 ± 4.6	32.0 ± 6.1	32.4 ± 5.1	32.1 ± 6.8	33.6 ± 5.1	28.6 ± 6.7
		(*p* = 0.023)		(*p* < 0.868)		(*p* = 0.028)
IPAQ	7,553 ± 7,454	6,314 ± 5,919	6,449 ± 5,451	6,188 ± 6,415	6,459 ± 5,059	5,900 ± 8,099
		(*p* = 0.347)		(*p* = 0.868)		(*p* = 0.805)
MSWS-12		26.4 ± 13.9	18.8 ± 9.7	34.5 ± 12.5	21.0 ± 10.3	42.0 ± 9.4
				(*p* < 0.001)		(*p* < 0. 001)
**Wear time of actiGraph and smartphone**						
Wear time ActiGraph minutes	4,498 ± 1,305	4,556 ± 1,692	4,056 ± 1,715	5,038 ± 1,547	4,638 ± 1,437	4,278 ± 2,407
		(*p* = 0.869)		(*p* = 0.036)		(*p* = 0.740)
Wear time smartphone minutes	3,684 ± 1,390	2,769 ± 1,980	2,783 ± 1,582	2,557 ± 2,315	2,884 ± 2,071	2,081 ± 1,742
		(*p* < 0.005)		(*p* = 0.733)		(*p* = 0.171)
**Smartphone outcomes**						
meanVM	9.86 ± 0.08	9.83 ± 0.07	9.83 ± 0.07	9.88 ± 0.07	9.85 ± 0.07	9.87 ± 0.09
		(*p* = 0.103)		(*p* = 0.007)		(*p* = 0.467)
varVM	0.485 ± 0.26	0.264 ± 0.22	0.430 ± 0.19	0.103 ± 0.10	0.311 ± 0.23	0.138 ± 0.16
		(*p* < 0.001)		(*p* < 0.001)		(*p* = 0.007)
**ActiGraph outcomes**						
meanVM	2,405 ± 714	2,286 ± 583	2,375 ± 580	2,205 ± 583	2,275 ± 592	2,319 ± 575
		(*p* = 0.347)		(*p* = 0.309)		(*p* = 0.805)
Daily MVPA	218 ± 80	216 ± 83	189 ± 77	240 ± 81	213 ± 82	223 ± 88
		(*p* = 0.869)		(*p* = 0.023)		(*p* = 0.791)
Steps/minute	13.5 ± 3.43	11.6 ± 2.80	12.1 ± 2.24	11.0 ± 3.17	11.8 ± 2.94	10.9 ± 2.24
		(*p* < 0.001)		(*p* = 0.165)		(*p* = 0.421)

*Values represent mean ± SD if not otherwise indicated. p-values for group comparison (patients vs. controls, RRMS vs. PP-/SPMS and EDSS groups) based on chi-square test for rates or Student's t-test. p-values are corrected p-values through Benjamini–Hochberg*.

*T25FW, Timed 25-Foot Walk; FTSTS, Five-Times Sit-to-Stand Test; TTW, 3-Meter Timed Tandem Walk; 2MWT, 2-Minute Walk Time; 6MWT, 6-Minute Walk Time; GLTEQ, Godin Leisure-Time Exercise Questionnaire; FAI, Frenchay Activity Index; IPAQ, International Physical Activity Questionnaire; MSWS-12, 12-Item MS Walking Scale; meanVM, mean vector magnitude; varVM, variance of vector magnitude; daily MVPA, daily moderate to vigorous physical activity*.

### Correlations Between Smartphone Metrics and ActiGraph

First, we were interested in analyzing the correlation between standard ActiGraph outcomes, and smartphone-derived metrics (see Table 3 and Figure e3). Among all metrics, varVM correlated best with ActiGraph steps/minute within all participants (rho = 0.44, *p* < 0.001). However, this association was mainly driven by the correlation in healthy controls (rho = 0.478, *p* < 0.001), while it was clearly weaker but still significant in pwMS (rho = 0.29, *p* = 0.022).

**Table 3:** Spearman rho rank correlations between ActiGraph and smartphone data.

		**Control**	**pwMS**
**Smartphone vs. actiGraph**			
Smartphone (varVM)	ActiGraph (steps/minute)	0.478****	0.288*
	ActiGraph (meanVM)	0.378**	0.201
	ActiGraph (daily MVPA)	0.169	−0.128
Smartphone (meanVM)	ActiGraph (steps/minute)	0.003	0.058
	ActiGraph (meanVM)	−0.077	0.173
	ActiGraph (daily MVPA)	−0.162	0.327**
**Within accelerometer itself**			
Smartphone (varVM)	Smartphone (meanVM)	0.102	−0.243
ActiGraph (steps/minute)	ActiGraph (meanVM)	0.828****	0.796****
ActiGraph (steps/minute)	ActiGraph (daily MVPA)	0.533****	0.325*
ActiGraph (meanVM)	ActiGraph (daily MVPA)	0.727****	0.443**

FDR corrected p-values: *p < 0.05, **p < 0.01, ****p < 0.0001.

### Correlations Between Accelerometer Outcomes, Clinical Performance-Based Measures, and PROMS

**Figure 4:**
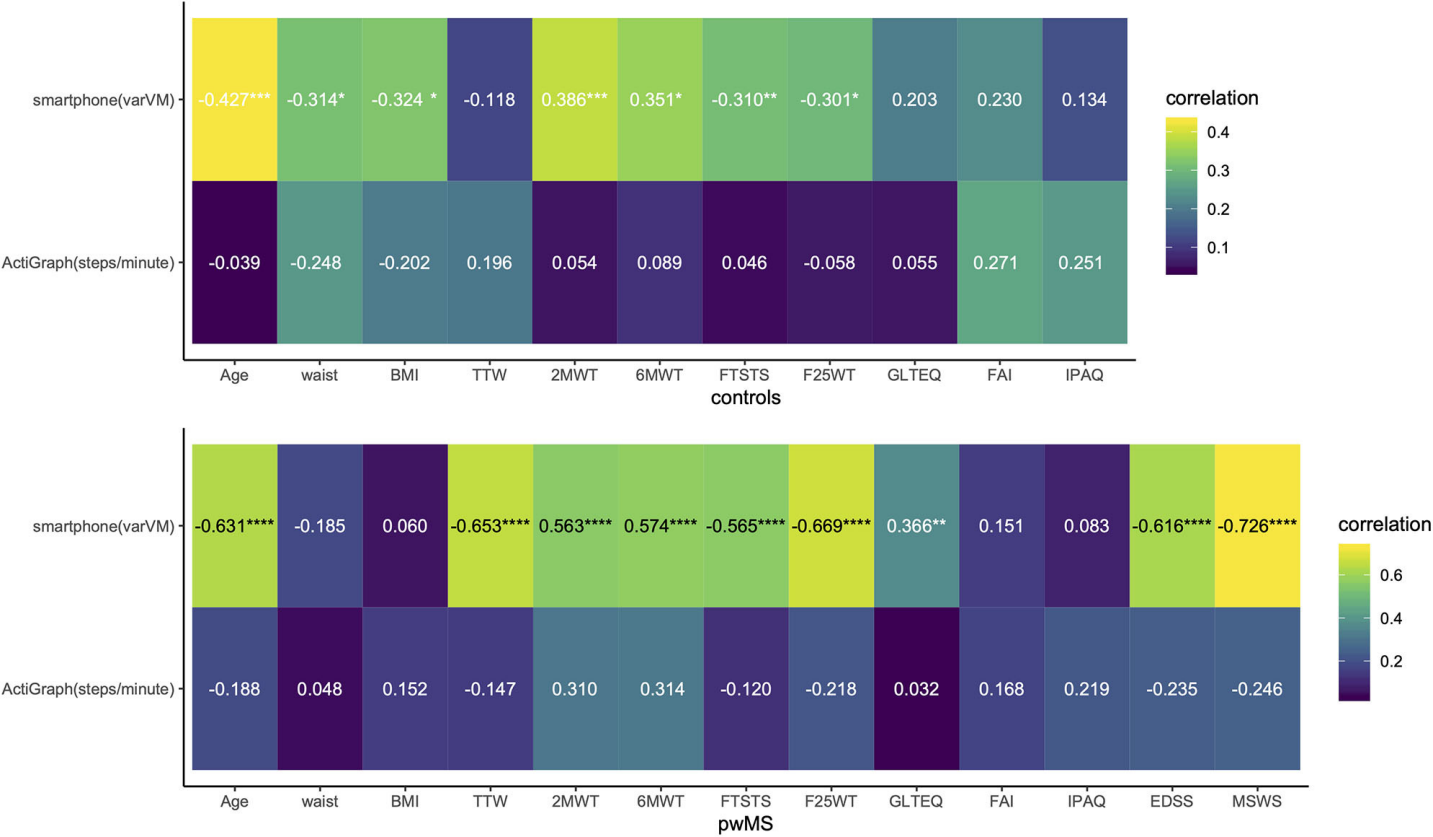
Association of clinical outcomes and questionnaires with smartphone varVM and ActiGraph steps/minute. Correlogram of Spearman rho; corrected *p*-values for multiple comparisons through FDR; *p < 0.05, **p < 0.01, ***p < 0.001, ****p < 0.0001.

Next, we investigated the association of both accelerometers with demography, clinical measures, and PROMS (Figure 4). Among the variables derived from ActiGraph, steps/minute showed within all participants the strongest but still only weak to moderate correlations with clinical measures (2MWT, 6MWT, FTSTS,T25FW rho = |0.21| to |0.34|, *p* < 0.05) and PROMS (FAI and IPAQ, rho = |0.27|, *p* < 0.05). In healthy controls and pwMS with ambulatory impairment, MVPA had a stronger association with some of the clinical measures than steps/minute (rho = |0.28| to |0.59|, *p* < 0.05). Among all variables derived from the smartphone, varVM showed the strongest correlations among all participants. varVM correlated mildly to moderately with the demography (age and waist, rho = |0.25| to |0.49|, *p* < 0.01), the clinical measures (TTW, 2MWT, 6MWT, FTSTS, T25FW, rho = |0.56| to |0.61|, *p* < 0.0001), and with PROMS (GLTEQ and FAI, rho = |0.39| and |0.25|, *p* < 0.01).

Thus, we will describe the smartphone outcome varVM and the ActiGraph outcome steps/minute as the most correlating outcomes in the subgroups more in detail. The association of both outcomes with demography, clinical measures, and PROMS are summarized in Figure 4 (for correlations of other accelerometer outcomes, see Supplemental Material). In healthy controls, varVM correlated moderately with most clinical measures (2MWT, 6MWT, F25WT, and FTSTS, rho = |0.30| to |0.39|, *p* < 0.05) but not with PROMs. Steps/minute did not correlate with any clinical measures nor with PROMs. Within all pwMS, varVM again moderately to strongly correlated with age (rho = −0.63, *p* < 0.0001), all clinical measures (TTW, 2MWT, 6MWT, FTSTS and T25FW, rho = |0.56| to |0.67|, *p* < 0.0001), GLTEQ (rho = 0.37, *p* < 0.01), EDSS (rho = −0.62, *p* < 0.0001), and MSWS-12 (rho = −0.73, *p* < 0.0001). Steps/minute did not, neither within all pwMS nor in the subgroups, correlate with any variable. Within minimal impaired pwMS, varVM showed moderate to strong correlation with age (rho = −0.74, *p* < 0.0001), clinical measures (TTW, 2MWT, 6MWT, FTSTS, and T25FW, rho = |0.45| to |0.64|, *p* < 0.01), EDSS (rho = −0.602, *p* < 0.0001), and MSWS-12 (rho = −0.74, *p* < 0.0001). Within ambulatory impaired subgroup, none of the accelerometer metrics correlated with any variable. Overall, varVM showed in comparison to steps/minute not only in the healthy subgroup but also in pwMS a stronger association with age, clinical measures, MSWS-12, and EDSS, representing walking ability and ambulatory impairment.

### The Ability of Accelerometry to Differentiate Between Subgroups

**Figure 5:**
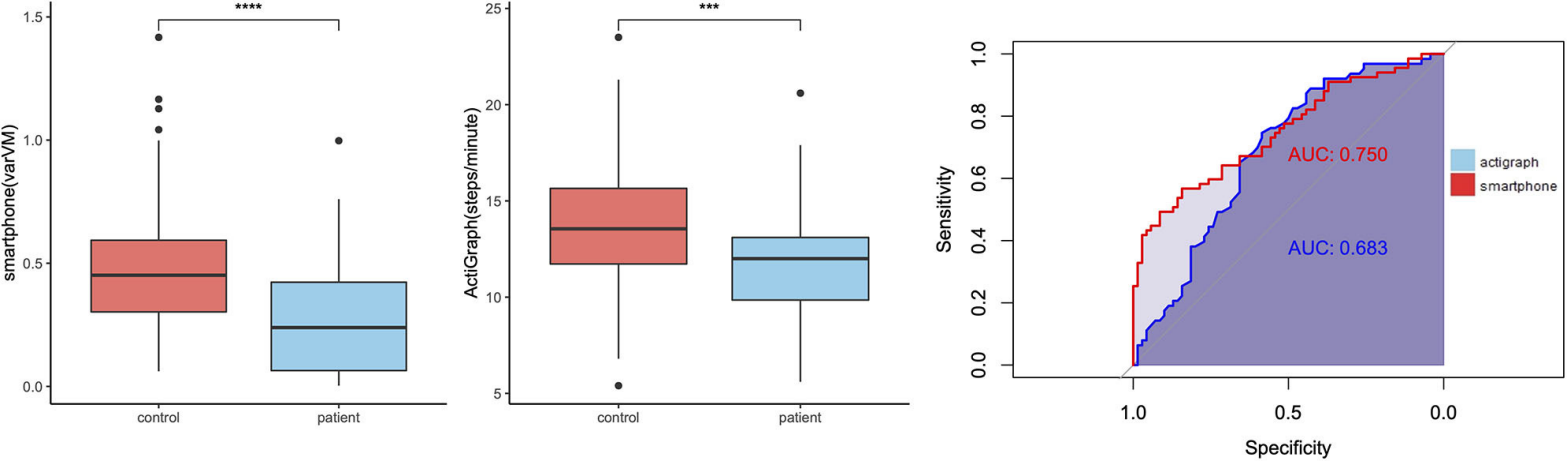
Group differences between healthy control and patients with multiple sclerosis (pwMS). Left: Boxplots showing smartphone and ActiGraph metrics for controls and pwMS, ***p < 0.001, ****p < 0.0001. Right: receiver operating characteristic (ROC) curves of smartphone (red) and ActiGraph (blue) showing the ability of differentiation between the groups.

**Figure 6:**
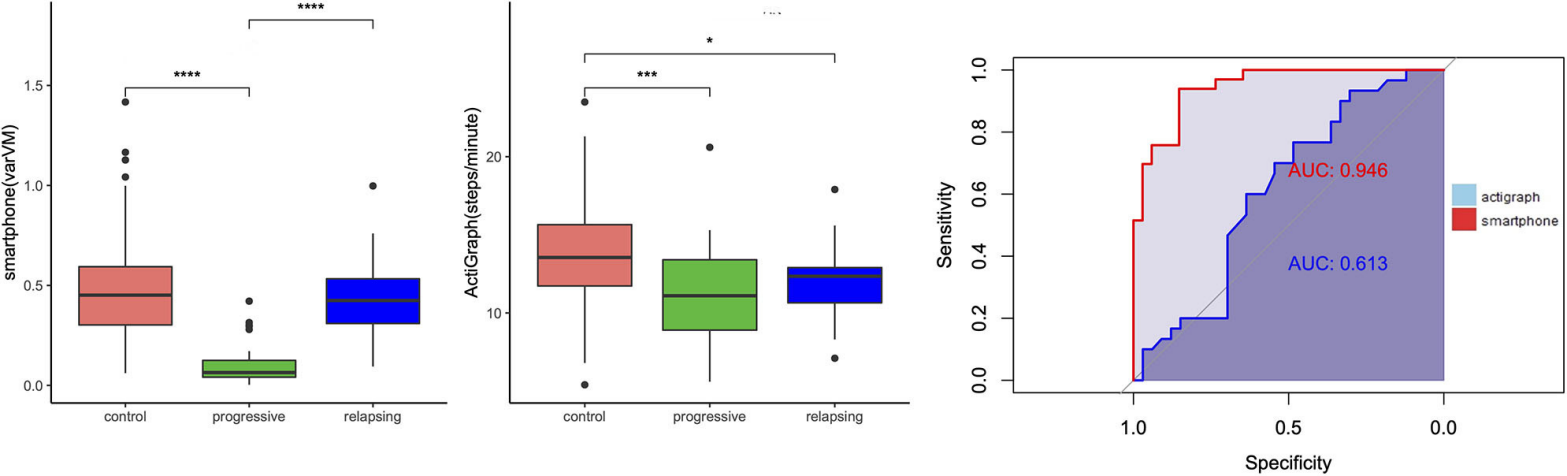
Group differences between relapsing–remitting and progressive multiple sclerosis (MS). Left: Boxplots showing smartphone and ActiGraph metrics for controls, progressive (or late) MS and relapsing (or early) MS. *p < 0.05, ***p < 0.001, ****p < 0.0001. Right: receiver operating characteristic (ROC) curves of smartphone (red) and ActiGraph (blue) showing the ability of differentiation between the MS groups.

**Figure 7:**
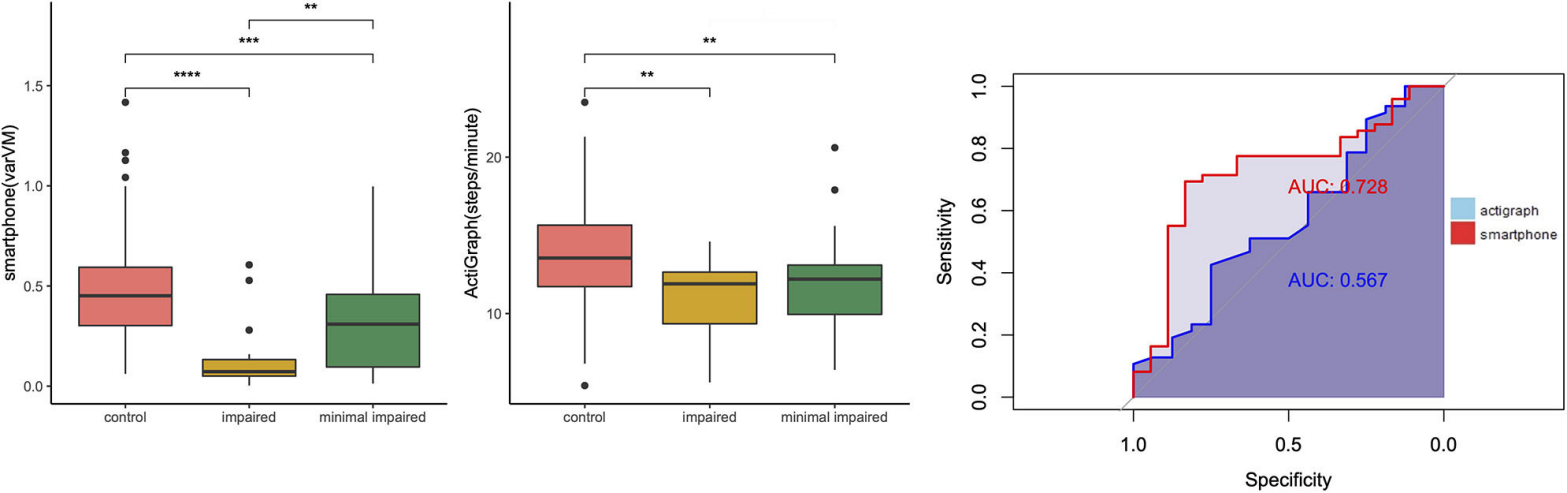
Group differences between ambulatory minimally impaired and ambulatory impaired patients with multiple sclerosis (pwMS). Left: Boxplots showing differences between controls minimally ambulatory impaired pwMS [Extended Disability Status Scale (EDSS) <3.5] and severely impaired pwMS (EDSS > 3.5), **p < 0.01, ***p < 0.001, ****p < 0.0001. Right: receiver operating characteristic (ROC) curves of smartphone (red) and ActiGraph (blue) showing the ability of differentiation of levels of ambulatory impairment.

In addition, we wanted to compare the discriminant abilities of accelerometry data for MS subgroups. Again, we used steps/minute and varVM as outcomes of interest. ROC analysis (Table 4) revealed that varVM was the better classifier for differentiating pwMS from control (AUC = 0.75 vs. 0.68, Figure 5). Moreover, only varVM was able to differentiate between relapsing–remitting and progressive MS (AUC = 0.946, *p* < 0.0001, Figure 6) and to differentiate between severe ambulatory impairment and mild ambulatory impairment patients (AUC = 0.728, *p* < 0.01, Figure 7).

**Table 4:** Sensitivity and specificity of accelerometry metrics to differentiate subgroups.

**Groups**	**Accelerometry**	**AUC**	**Sensitivity**	**Specificity**	**NPV**	**PPV**	**Delong's test *p*-value**
Control vs. patient	ActiGraph steps/minute	0.683	0.746	0.586	0.618	0.719	*p* = 0.286
	Smartphone varVM	0.750	0.567	0.843	0.776	0.670	
RRMS vs. PP-SP/MS	ActiGraph steps/minute	0.613	0,767	0.484	0.696	0.575	*p* < 0.001
	Smartphone varVM	0.946	0.939	0.853	0.935	0.861	
Impaired vs. minimal impaired	ActiGraph steps/minute	0.567	0.426	0.750	0.308	0.833	*p* = 0.153
	Smartphone varVM	0.728	0.694	0.833	0.500	0.919	

*ROC analyses. The area under the curve (AUC) results were compared with Delong's test*.

*AUC, area under the curve; NPV, negative predictive value; PPV, positive predictive value*.

## Discussion

This study examined smartphone accelerometry as an outcome of real-life ambulation and physical activity in healthy individuals and pwMS. To follow this aim, we analyzed the relationship of putative smartphone metrics with a research-grade accelerometer (ActiGraph) during free-living conditions, with objectively measured walking ability and with self-reported physical activity. Overall, results showed that the smartphone accelerometer correlated only moderately with ActiGraph in HC and pwMS. However, the smartphone accelerometer seems to be more closely associated with walking ability, represented by the clinical performance-based measures, such as TTW, T25FW, 2-/6-MWT, FTSTS, and with ambulatory impairment, represented by MSWS and EDSS. Moreover, the smartphone accelerometer differentiated the levels of ambulation among all participants and ambulatory impairment among the pwMS better than the ActiGraph. In our study, smartphone metrics seem more reliable than a wrist-worn research-grade accelerometer.

For this study, we used a new metric to capture ambulation and body motion based on accelerometry data—the variance of the vector magnitude. From a conceptual point of view, the metric represents the movement of the smartphone in all dimensions in a given time. The metric was chosen based on a technical validation course and provided two important features: high specificity and sensitivity to identify wear time periods and a positive association with increased ambulation. An advantage of this metric is its independence from the orientation of the smartphone. The value of this metric was evaluated in comparison to a battery of different outcomes and was chosen by applying a strict selection methodology based on an explorative and a validation data set. Moreover, the promising results of being a good discriminator between ambulation levels and its association with ambulatory impairment metrics indicate a successful proof-of-concept.

The rather weak to moderate association between smartphone varVM and ActiGraph outcomes in both HC and pwMS contrasts one study that android smartphones provided similar raw counts as ActiGraph in a free-living setting (29). Although ActiGraph is a validated tool, most of those validating studies chose the hip-worn position (20, 21), and the literature provides controversial data for the wrist-worn position of accelerometers (12, 23, 38–40). However, the acceptance for the wrist-worn position may be higher (39). In this study, we also used wrist-worn ActiGraph data, which might explain the unexpected low to moderate correlations with clinical measures and PROMS. Another reason for the lower correlation could be explained by the arm movements during the non-walking time included in the high active wear time, while the participants might move the smartphone mostly when they were walking.

Regarding the ecological validity, ActiGraph could influence the exercising behavior and, for example, increase the physical activity, since the notable visibility and discomfort on the wrist could be perceived invasive as a “reminder.” On the other hand, the possibly perceived invasiveness of a wrist-worn accelerometer could be intentionally used to motivate users for more exercising. Eventually, using smartphones as an ubiquitous, available measuring tool might overcome these shortcomings as usual smartphone positions such as handbags, rucksacks, or pants pocket, which are less perceivable and provide a high accuracy (40, 41). These positions are closer to the body's center of mass that has been recommended as the best sensor position (42). Thus, smartphones could refer more to real life and offer higher ecological validity. However, the perceived invasiveness was not studied here and needs to be addressed in future studies.

An important result supporting the value of our smartphone-based approach is the clear association with age. Walking abilities, and especially walking speed, are known to be an important indicator of health status and associated with age (43). Interestingly, this important coherence with age was well-reproducible with our smartphone metric but not with the ActiGraph. Proving a well-known and fundamental relationship emphasizes the reliability of smartphone accelerometry as an outcome of real-life ambulation. Furthermore, the discriminating ability of smartphone varVM confirmed the assumption that smartphones could differentiate levels of walking ability and ambulatory dysfunction. However, these findings are based on a cross-sectional study, and its sensitivity to disability progression or improvement must be analyzed in a longitudinal setting.

However, it remains uncertain which dimension of physical activity or ambulation is captured explicitly by smartphones in general: There are controversial results addressing this issue in the literature of former research (27, 29, 44). Here, our approach using a research-grade accelerometer as a reference failed. However, varVM correlated much stronger with the clinical measures, representing walking ability than with the PROMs and representing self-reported physical activity. Thus, we assume that smartphone measures rather the walking ability than the physical activity. It might be due to a measurement gap during exercising or other vigorous activities performed without the smartphone. Conceptually, this assumption is supported by the fact that smartphones are usually worn during habitual activities like traveling, shopping, and walking outside. At the same time, it is preferably placed aside during exercising and other vigorous activities. However, this assumption needs further investigation.

The association between smartphone metrics and clinical outcomes was, in general, higher than for the ActiGraph. However, both smartphone and ActiGraph correlated with clinical measures or PROMs more among the ambulatory mildly to moderately impaired pwMS than those with severely impaired ambulation. This links to the still open question of whether accelerometry can generally measure walking ability or rather physical activity in patients with very low activity levels and variable gait patterns, such as in progressive MS. However, the ability of the clinical test to mirror real-life ambulation and motion is also limited, and they have a rather low ecological validity (15, 45). Thus, a poor association might be due to the low performance of the real-life device or shortcomings of the clinical tests. Further research is needed to provide better objective estimates of low activity levels in more severely disabled patients. Moreover, our technical validation indicated a meaningful increase in the chosen smartphone metric with increasing physical activity. However, these findings could not be validated in the real-life setting in this study.

One of the limitations in our study was the wear time of the devices that might have been too short for reliable estimates of real-life walking or activity. The original wristlet of ActiGraph was often reported as unfeasible during specific exercising like weightlifting; on the other hand, the smartphone has a relatively short battery life and needed to be charged at least once a day. Wear time alone cannot be considered as evidence for the smartphone as an outcome of real-life activity. However, smartphone covered ~72% of the ActiGraph measurement time. Future studies need to validate against other outcomes or devices. Moreover, we asked the participants to wear the smartphone in their habitual wearing position, aiming to simulate the real-life condition and to avoid the possibly perceived invasiveness. Although the usual position like handbag, backpack, and pants pocket probably does not have differences in the accuracy of measurement (40), the smartphone secured on the upper arm showed a lower accuracy (41). Another limitation is that it is impossible to determine if the phone estimates would remain comparable with other phone models that have not been tested. However, one of the most prominent android brands was used in this study. Finally, we only investigated a rather simple summary metric of 60-s epochs, which reduces the complexity of the raw data. Advanced algorithms, for example, estimating walking speed, might improve the validity of smartphone accelerometry, as it has been shown for research-grade devices (25).

Even with these limitations, there seems to be a strong opportunity for smartphone accelerometry in the context of several diseases and healthy living (27, 30). It might help clinicians to monitor ambulatory dysfunction, disease progress, or rehabilitation in diverse clinical conditions with high ecological validity. It could also help patients to monitor their individual changes of walking ability from a personal baseline over time and to achieve ability goals. Combined with motivational, educational tools, it may as well help to improve physical activity independent from diseases.

## Conclusion

Smartphone accelerometry provides better estimates of mobility and disability than a wrist-worn standard accelerometer in a free-living context for both controls and pwMS. Given the fact that no additional device is needed and despite further validation, smartphone accelerometry might be a convenient outcome of real-life ambulation in healthy individuals and chronic diseases such as MS. Moreover, activity estimates from smartphones might be more ecological valid as the perceived invasiveness of assessment is lower than for additional and clearly visible devices.

## Data Availability Statement

The datasets generated for this study are available on request to the corresponding author.

## Ethics Statement

The studies involving human participants were reviewed and approved by Ärztekammer Hamburg. The patients/participants provided their written informed consent to participate in this study.

## Author Contributions

YZ: design and conceptualized study, major role in the acquisition of data, analyzed the data, and drafted the manuscript for intellectual content. NN: major role in the acquisition of data and revised the manuscript for intellectual content. JP: interpreted the data and revised the manuscript for intellectual content. EG: major role in the acquisition of data, interpreted the data, and revised the manuscript for intellectual content. CH: interpreted the data and revised the manuscript for intellectual content. J-PS: design and conceptualized study, analyzed the data, and drafted the manuscript for intellectual content. All authors contributed to the article and approved the submitted version.

## Conflict of Interest

JP reports grants from Deutsche Rentenversicherung Bund and from MerckSerono outside the submitted work. CH reports grants and personal fees from Biogen, Genzyme, MerckSerono, Novartis, Roche outside the submitted work. J-PS receives research funding from Deutsche Forschungsgemeinschaft and reports a grant from Biogen that partially funded the submitted work. A further grant is reported from Genzyme outside the submitted work. J-PS received travel support and personal fees from Alexion, Biogen, and Genzyme outside the submitted work. The remaining authors declare that the research was conducted in the absence of any commercial or financial relationships that could be construed as a potential conflict of interest.
