# Supplementary material for: Smartphone Accelerometry: A Smart and Reliable Measurement of Real-Life Physical Activity in Multiple Sclerosis and Healthy Individuals
Source: Front Neurol. 2020 Aug 14;11:688. doi: 10.3389/fneur.2020.00688 (PMC7456810; doi:10.3389/fneur.2020.00688)
Supplement: Supplementary file 1 [file Presentation_1.PDF]

Supplemental material

Figure e1 – No wear vs wear: Boxplots of putative smartphone outcomes in the explorative technical validation subset

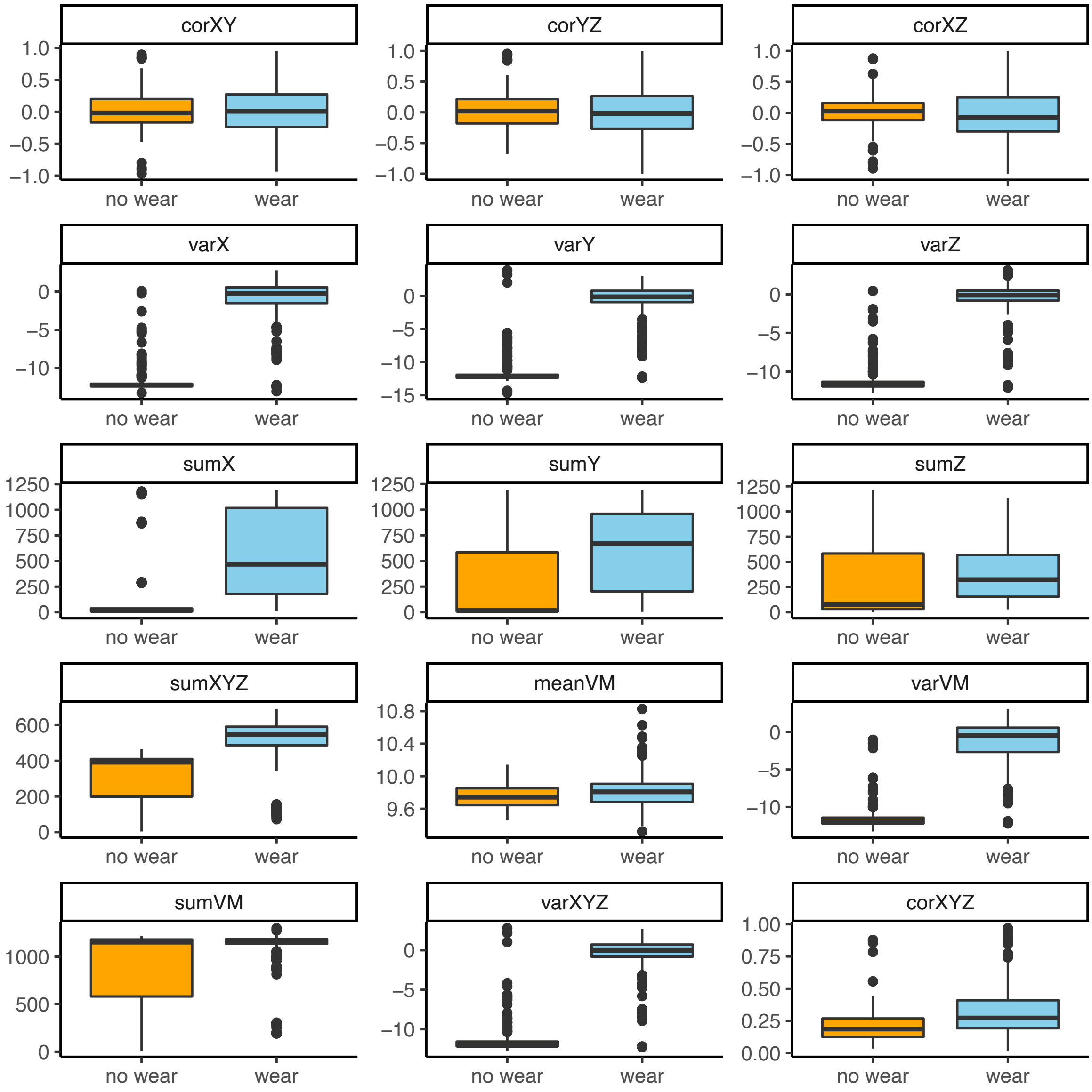

Boxplots comparing wear and no wear periods of 28 smartphones: Sum of absolute axis values (sumX, sumY, sumZ), variance of axis values (varX, varY, varZ), Pearson's correlations between each pair of axes (corXY, corXZ, corYZ), sum of all absolute axis values (sumXYZ), mean absolute correlation (corXYZ), sum of absolute vector magnitude (sumVM), mean vector magnitude (VM) and mean variance of the vector magnitude (varVM).

Figure e2 – Activities: Boxplots of putative smartphone outcomes in the explorative technical validation subset

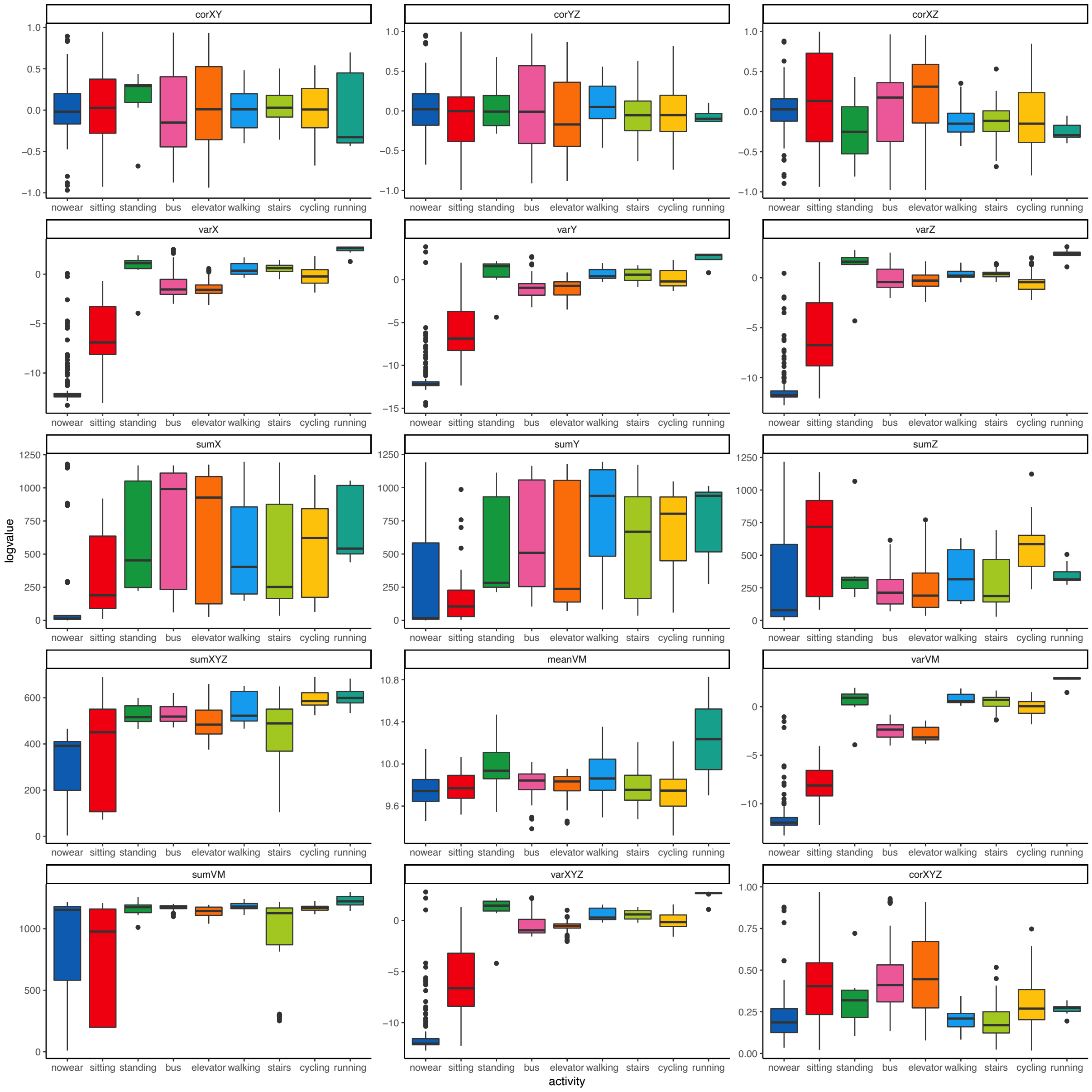

Boxplots comparing periods of different activities of 28 smartphones: Sum of absolute axis values (sumX, sumY, sumZ), variance of axis values (varX, varY, varZ), Pearson's correlations between each pair of axes (corXY, corXZ, corYZ), sum of all absolute axis values (sumXYZ), mean absolute correlation (corXYZ), sum of absolute vector magnitude (sumVM), mean vector magnitude (VM) and mean variance of the vector magnitude (varVM).

Figure e3 - Association between ActiGraph(steps/minute) and smartphone (variance of vector magnitude/varVM) in subgroups

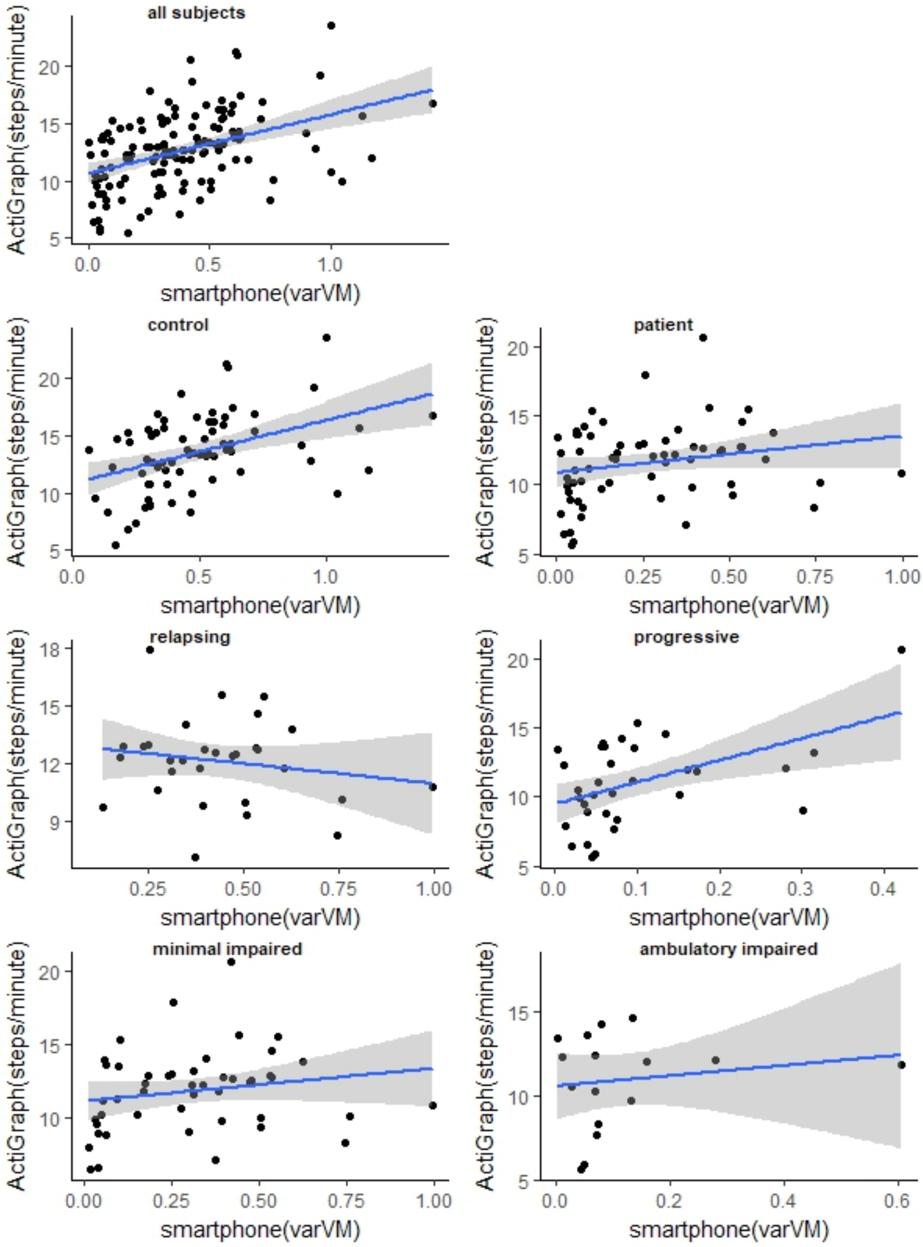

Table e1 - Correlations between accelerometry data and clinical outcomes

All subjects											
	age	waist	BMI	TTW	2MWT	6MWT	FTSTS	F25WT	GLTEQ	FAI	IPAQ
smartphone											
varVM	-0.487****	-0.254**	-0.104	-0.565****	0.566****	0.586****	-0.583****	-0.605****	0.385****	0.252**	0.169
meanVM	-0.001	-0.128	-0.131	0.314***	-0.096	-0.136	0.176*	0.251**	-0.085	-0.112	-0.030
ActiGraph											
Steps/ minute	-.0104	-.0137	-0.054	-0.150	0.294***	0.337***	-0.211*	-0.271**	0.144	0.272**	0.274**
meanVM	-0.027	-0.172*	-0.133	0.019	0.130	0.186*	-0.045	-0.128	-0.019	0.193*	0.244**
Daily MVPA	0.316***	-0.109	-0.076	0.191*	-0.104	-0.011	-0.085	0.105	-0.035	0.259**	0.190*

Spearman rho correlations; * p-value < .05, ** p-value <.01, *** p-value <.001, **** p-value <.0001

Table e2 - Spearman rho correlations between accelerometers and clinical outcomes within control

Only controls											
	age	waist	BMI	TTW	2MWT	6MWT	FTSTS	F25WT	GLTEQ	FAI	IPAQ
smartphone											
Variance of vector magnitude	-0.427***	-0.314**	-0.324**	-0.118	0.386***	0.351**	-0.310**	-0.301*	0.203	0.230	0.134
Mean vector magnitude	-0.217	-0.256*	-0.187	0.121	0.060	0.031	-0.109	0.086	0.135	-0.225	-0.020
ActiGraph											
Steps/minute (ActiGraph)	-0.039	-0.248*	-0.202	0.196	0.054	0.089	0.046	-0.058	0.055	0.271*	0.251*
mVM	0.049	-0.208	-0-208	0.207	0.026	0.078	0.106	-0.056	-0.070	0.271*	0.223
Daily MVPA(ActiGraph)	0.396***	-0.106	-0.076	0.326**	-0.276*	-0.172	0.203	0.210	-0.037	0.322**	0.277*

* p-value < .05, ** p-value <.01, *** p-value <.001, **** p-value <.0001

Table e3 - Spearman rho correlations between accelerometers and clinical outcomes within pwMS

Only patients													
	age	waist	BMI	TTW	2MWT	6MWT	FTSTS	F25WT	GLTEQ	FAI	IPAQ	EDSS	MSWS
smartphone													
Variance of vector magnitude	-0.631****	-0.185	0.060	-0.653****	0.563****	0.574****	-0.565****	-0.669****	0.366**	0.151	0.083	-0.616****	-0.726****
Mean vector magnitude	0.283*	-0.086	-0.110	0.353**	-0.080	-0.106	0.143	0.310*	-0.226	0.141	0.034	0.242*	0.154
ActiGraph													
Steps/minute (ActiGraph)	-0.188	0.048	0.152	-0.147	0.310*	0.314*	-0.120	-0.218	0.032	0.168	0.219	-0.235	-0.246
mVM	-0.132	-0.139	-0.049	-0.006	0.191	0.214	-0.112	-0.120	-0.002	0.073	0.281*	-0.111	-0.118
Daily MVPA(ActiGraph)	0.250*	-0.108	-0.063	0.244	0.045	0.085	0.067	0.061	-0.049	0.198	0.124	0.182	0.123

* p-value < .05, ** p-value <.01, *** p-value <.001, **** p-value <.0001

Table e4 - Spearman rho correlations between accelerometers and clinical outcomes within persons with relapsing MS

Persons with relapsing MS													
	age	waist	BMI	TTW	2MWT	6MWT	FTSTS	F25WT	GLTEQ	FAI	IPAQ	EDSS	MSWS
smartphone													
Variance of vector magnitude	-0.401*	0.140	0.259	-0.392*	0.269	0.365*	-0.302	-0.304	0.211	0.293	0.063	-0.230	0.546**
Mean vector magnitude	0.073	-0.070	-0.038	0.135	0.003	0.144	-0.171	0.044	-0.167	0.011	0.287	-0.044	-0.106
ActiGraph													
Steps/minute (ActiGraph)	0.173	0.049	-0.003	0.326	0.197	0.196	-0.000	-0.025	-0.045	0.071	0.139	-0.127	0.105
mVM	0.139	-0.180	-0.142	0.201	0.189	0.245	-0.249	-0.045	-0.126	-0.104	0.278	-0.017	0.203
Daily MVPA(ActiGraph)	0.079	-0.148	-0.144	0.050	0.356	0.423*	-0.333	-0.317	0.95	-0.043	0.106	0.198	0.071

* p-value < .05, ** p-value <.01, *** p-value <.001, **** p-value <.0001

Table e5 - Spearman rho correlations between accelerometers and clinical outcomes within persons with progressive MS

Persons with progressive MS													
	age	waist	BMI	TTW	2MWT	6MWT	FTSTS	F25WT	GLTEQ	FAI	IPAQ	EDSS	MSWS
smartphone													
Variance of vector magnitude	-0.213	-0.111	0.031	-0.156	0.310	0.278	-0.095	-0.310	0.162	0.104	-0.099	-0.260	-0.278
Mean vector magnitude	0.083	-0.355*	-0.230	0.280	0.090	-0.039	0.037	0.182	-0.132	0.200	-0.040	0.173	-0.131
ActiGraph													
Steps/minute (ActiGraph)	-0.288	0.292	0.296	-0.143	0.233	0.270	0.021	-0.138	0.015	0.201	0.248	-0.098	-0.364*
mVM	-0.203	0.093	0.083	0.085	0.116	0.164	0.136	-0.042	0.002	0.165	0.263	0.018	-0.276
Daily MVPA(ActiGraph)	0.062	-0.198	-0.032	0.106	0.190	0.244	-0.027	-0.136	-0.014	0.445*	0.243	-0.236	-0.354

* p-value < .05, ** p-value <.01, *** p-value <.001, **** p-value <.0001

Table e6 - Spearman rho correlations between accelerometers and clinical outcomes within EDSS < 3.5

pwMS with minimal ambulatory impairment													
	age	waist	BMI	TTW	2MWT	6MWT	FTSTS	F25WT	GLTEQ	FAI	IPAQ	EDSS	MSWS
smartphone													
Variance of vector magnitude	-0.736****	-0.246	-0.80	-0.643****	0.445**	0.503***	-0.640****	-0.611****	0.221	-0.042	0.005	-0.602****	-0.740****
Mean vector magnitude	0.335*	0.104	0.133	0.253	-0.071	-0.090	0.189	0.308*	-0.282	0.264	0.124	0.203	0.108
ActiGraph													
Steps/minute (ActiGraph)	-0.103	-0.061	0.067	-0.088	0.241	0.236	-0.107	-0.167	-0.117	0.022	0.156	-0.243	-0.152
mVM	-0.135	-0.171	-0.133	-0.068	0.267	0.286	-0.270	-0.207	-0.098	-0.017	0.225	-0.242	-0.173
Daily MVPA(ActiGraph)	0.397**	-0.097	-0.095	0.390**	-0.022	0.064	0.193	0.178	-0.125	0.125	0.152	0.329*	0.252

Table e7 - Spearman rho correlations between accelerometers and clinical outcoms within EDSS >= 3.5

pwMS with ambulatory impairment													
	age	waist	BMI	TTW	2MWT	6MWT	FTSTS	F25WT	GLTEQ	FAI	IPAQ	EDSS	MSWS
smartphone													
Variance of vector magnitude	-0.088	-0.150	0.135	-0.021	0.298	0.326	0.098	-0.480*	0.169	0.232	-0.039	-0.553*	-0.205
Mean vector magnitude	0.133	-0.468	-0.525*	0.318	0.097	0.061	0.042	0.370	0.037	0.034	0.068	0.359	-0.031
ActiGraph													
Steps/minute	-0.346	0.295	0.459	-0.235	0.460	0.564*	0.025	-0.188	0.409	0.538*	0.264	-0.368	-0.639*
mVM	-0.116	-0.038	0.038	0.103	-0.047	0.068	0.379	0.174	0.474	0.697**	0.621*	-0.219	-0.646*
Daily MVPA	-0.097	-0.183	-0.050	-0.240	0.550*	0.593*	-0.186	-0.238	0.345	0.562*	0.280	-0.558*	-0.407

* p-value < .05, ** p-value <.01, *** p-value <.001, **** p-value <.0001
